# The association between tibial torsion, knee flexion excursion and foot progression during gait in people with knee osteoarthritis: a cross-sectional study

**DOI:** 10.1186/s13102-023-00726-z

**Published:** 2023-09-13

**Authors:** Chen Huang, Ping-Keung Chan, Kwong-Yuen Chiu, Chun-Hoi Yan, Shun-Shing Yeung, Christopher Wai-Keung Lai, Aaron Kam-Lun Leung, Siu Ngor Fu

**Affiliations:** 1 Department of Rehabilitation Sciences, The Hong Kong Polytechnic University, Hong Kong, China; 2 Department of Orthopaedics and Traumatology, Queen Mary Hospital, The University of Hong Kong, Hong Kong, China; 3 Physiotherapy Department, MacLehose Medical Rehabilitation Centre, Hong Kong, China; 4 Health and Social Sciences Cluster, Singapore Institute of Technology, Singapore, Singapore

**Keywords:** Knee osteoarthritis, Tibial torsion, Gait kinematics, Osteoarthritis

## Abstract

**Background:**

Lower limb malalignment is associated with gait kinematics, but there is limited information on the relationship between gait kinematics and tibial torsion in individuals with knee osteoarthritis (OA). This study aimed to investigate possible associations between tibial torsion and early stance kinematics during gait in people with mild and moderate medial knee OA.

**Methods:**

Forty-seven participants (age: 62.1 ± 6.0 years; female/male: 37/10) diagnosed with medial knee OA were recruited from a regional hospital. Thirty of them had mild and seventeen had moderate knee OA. Lower limb alignment including tibial torsion and valgus/varus alignment were assessed by an EOS biplaner X-ray system with participants in weight-bearing position. Lower limb kinematics during gait was captured using the Vicon motion analysis system. The associations were estimated by partial Pearson correlation coefficient test.

**Results:**

Our results indicated that external tibial torsion was related to early stance knee flexion excursion in participants with moderate knee OA (*r* = -0.58, *p* = 0.048), but not in participants with mild knee OA (r = 0.34, *p* = 0.102). External tibial torsion was associated with external foot progression angle (*r* = 0.48, *p* = 0.001), and knee varus/valgus alignment was associated with knee flexion excursion (*r* = -0.39, *p* = 0.010) in all participants.

**Conclusions:**

Both horizontal and frontal lower limb alignments were associated with knee flexion excursion at early stance of gait cycle in participants with medial knee OA. The distal rotational profile of lower limb would likely affect knee motion in sagittal plane. It implies that people with moderate knee OA could possibly benefit from correction of rotational alignment of lower limb.

## Background

Osteoarthritis (OA) is a common chronic condition affecting more than 300 million people worldwide [1]. Knee OA is characterized by pain, knee joint stiffness, and limited knee joint range of motion. Common risk factors associated with knee OA include age, gender, malalignment, and other medical conditions [2, 3].

Altered gait kinematics at the knee joint and lower limb have been reported in individuals with knee OA. Specifically, individuals with knee OA have been observed to exhibit greater knee flexion at heel strike, reduced knee flexion excursion during early stance [4, 5], and altered foot progression angle [6, 7] during gait. Although research shows that altered sagittal plane knee kinematics [8] and foot progression angle [9] during gait was associated with joint loading, little is known on the mechanical factors that play a role in the development of these altered gait kinematics.

From a mechanical perspective, joint motion is determined by joint architecture. Malalignment of the lower limb might be an important factor that affects knee joint kinematics during gait. Tibial torsion, as a critical component of torsional alignment of the lower limb, was altered in those with knee OA. Smaller external tibial torsion was detected in people with knee OA than healthy controls [10]. Torsional malalignment of the lower limb is one of the factors in relation to abnormal gait rotational changes [11]. Knee joint has a locking mechanism: when it moves from 30° knee flexion to fully extension (0^°^), a torsional motion is associated [12]. During the loading response of the stance phase, knee joint moves from extension to flexion with an associated external torsional motion on the tibia, which is known as the unscrew-home mechanism the knee joint [13]. This coupling mechanism is disrupted in people with medial knee OA [14]. It implies an interaction between torsional alignment and sagittal motion. Knee flexion–extension motion in early stance phase could be altered due to tibial torsion in people with knee OA. A computerized model study demonstrated that tibial torsion could affect activation of lower limb muscles and influence knee flexion–extension movement [15]. While studies have shown that tibial torsion can affect knee flexion motion, the exact nature of this relationship is not fully understood, especially in people with knee OA. External tibial torsion was also associated with abnormal foot progression angle in pediatric patients waiting for surgery [16]. Whether similar relationship happens in senior with knee OA has not been investigated. Such information is important because foot progression angle was associated with joint loading. The limited understanding of the complex biomechanical interactions between tibial torsion and lower limb kinematics during gait constrains the treatment, and prevention of related conditions in knee OA.

Aside from alignment, the presence of marginal osteophytes and joint space narrowing, which are the determinants of radiographic severity of knee OA, could restrict both passive and active knee range of motion [17]. Thus, the relationship between lower limb alignments and knee flexion motion during early stance phase of gait might be affected by these differences in bone and/or joint structures in people with different severities of knee OA. It is essential to explore the impact of disease severity since there is a possible effect of structural changes on gait kinematics. Findings from this study would enhance our understanding on the mechanical loading in people with knee OA.

## Methods

### Aims

The aims of this study were to (1) delineate the relationships between lower limb alignments and lower limb kinematics at early stance phase during gait; (2) explore whether the relationship would differ in individuals with mild and moderate knee OA. Our hypotheses were (1) both knee varus and tibial torsion would be associated with lower limb kinematics; (2) the associations would be specific to radiographic severity of knee OA.

### Study design

This was a cross-sectional observational study.

### Participants

Participants were recruited a local knee osteoarthritis management program from the Department of Orthopedic and Traumatology of Queen Mary Hospital, Hong Kong. The following inclusion criteria were: 1) aged between 50 and 80 years, 2) having radiographic evidence of OA in the medial compartment of tibiofemoral joint with Kellgren-Lawrence (KL) grading < 4, 3) pain score ≥ 2 on an 11-point visual analog scale (VAS) in the past month. Patients were excluded if they had any of the followings: 1) more osteophytes in the lateral compartment, 2) intra-articular injection within the past 6 months, 3) rheumatoid arthritis, 4) history of surgery to either knee joint, 5) other conditions influencing lower limb functions, 6) any pain of lower back or lower limb joint with intensity ≥ 3 on VAS, 7) unable to walk without assistance and, 8) body mass index (BMI) > 36 kg/m^2^ [18]. Radiographic imaging was rated by experienced orthopedic surgeons for KL grading. Radiographic findings were used to categorize the disease severity as mild (KL grade 1 and 2) and moderate (KL grade 3) [19]. The sample size was estimated according to findings of a pilot study investigating the relationship between pain and tibial torsion. With an effect size of 0.43, the sample size needed would be 38 to obtain an 80% power with α = 0.05.

This study was approved by the Human Subjects Ethics Committee of the administrating institution (HSEARS20170406003). All participants gave their informed written consent at the beginning of the study.

### Outcome measurements

All the outcome measurements were finished before the start of any interventional management. The assessments were conducted in laboratory settings in a regional hospital.

#### Joint alignments

Static joint alignments including tibial torsion and knee varus angle were measured with an EOS biplanar low-dose X-ray imaging system (EOS imaging, Paris, France). To ensure clear bony structure recognition, all participants adopted a standing position with right foot shifting forward for around 4 cm [20]. An experienced researcher who underwent two-week training by a certificated radiologist conducted all the alignment measurements. The three-dimensional reconstruction from biplanar x-ray images was performed with sterEOS (Version 1.6, EOS imaging, Paris, France). Anatomical reference points were identified manually by the assessor including femoral head, neck, greater/lesser trochanters, intercondylar notch, lateral and medial condyles, tibial spines, lateral and medial tibial plateau, distal tibial articular surface, and medial malleolus. Fine adjustment of bony contours was operated according to the EOS guideline, and statical joint alignments were exported automatically by the sterEOS software [21]. Tibial torsion was determined by posterior tibial plateau tangent axis and bi-malleolar axis. Positive value was defined as external rotation of bi-malleolar plane to tibial plateau plane and negative value as internal rotation. Knee varus angle was measured as the angle between the femoral mechanical axis and the tibial mechanical axis. Higher positive value indicated larger knee varus angle. The EOS x-ray imaging system had great agreement with standard CT measurements which was considered as the gold standard in measuring tibial torsion [20]. Excellent intra-rater inter-day reliability was obtained using two-way mixed model intraclass correlation coefficient (ICC). The two measurements were conducted on two different days with a one-week interval (ICC_tibialtorsion_ = 0.93, ICC_kneevarusangle_ = 0.99).

#### Lower limb kinematics during gait

Lower limb kinematics information was captured with a Vicon motion analysis system (Vicon Motion Systems Ltd, Oxford, UK) of a total of eight cameras (Vicon, Model MX T40). Two floor-mounted force plates (Kistler Group, Winterthur, Switzerland) were used to detect heel strikes in order to deciding events of gait cycle. The kinematic and kinetic data were recorded at a sampling rate of 100 Hz and 1000 Hz respectively. The Vicon Plug-In-Gait Lower body model was used for modelling and the marker set of bilateral anterior superior iliac spine, posterior superior iliac spine, thigh, knee, tibia, ankle, toe and heel was settled on each participant by an experienced gait laboratory technician according to the guide of the manufacturer [22]. Participants were instructed to hold standstill in a neutral position for static calibrations. They were invited to walk barefoot for 8 m at comfortable speed. Participants were allowed for sufficient practice trials, and they had to finish five successful trials of each lower limb with clean heel strike and toe off on force plates. The Vicon system is considered one of the gold standard in optoelectronic motion capture industry [23].

Lower limb sagittal kinematics during early stance phase was extracted with Vicon Nexus software (Version 2.5, Vicon Motion Systems Ltd, Oxford, UK). Butterworth filter of 6 Hz was used for processing kinematic data and 15 Hz for kinetic data. The rigid body segments of pelvis, thigh, shank and foot were estimated by the Vicon Plug-In-Gait model [24]. The events of heel strike and toe off were decided by a combination of force plate data and foot marker trajectories to improve the accuracy detection. Knee flexion, ankle dorsi-flexion and foot progression angle at initial contact were calculated. Peak knee flexion and ankle dorsi-flexion angle during early stance phase was identified as the maximum angle between initial contact and loading response. Ankle dorsi-flexion and knee flexion excursion was defined as the knee flexion–extension range respectively between the angle at initial contact and the peak angle. Positive values for knee, ankle and knee indicated knee flexion, ankle dorsi-flexion and external foot progression respectively.

### Statistical analysis

SPSS (Version 23.0, IBM Corp., New York, US) was used for data analysis. Normality of data was assessed with Shapiro–Wilk test. Correlations between each lower limb kinematic in early stance phase and alignments were assessed by partial Pearson’s correlation coefficient test controlling for gender, age, walking speed, pain and BMI. How radiographic severity of OA interacted with the relationships between kinematics and alignment was examined with linear regression with interaction. The assumptions of homoscedasticity and normality of linear regression were assessed accordingly. When significant interaction was revealed, subgroup analysis of partial correlation was conducted in different levels of radiographic severity of OA. Statistically significant level was set at 0.05. There was no missing data in the current study.

## Results

Out of 100 potential participants with knee OA admitted between March 2017 and July 2018, forty-seven were recruited after screening for selection criteria. Most exclusion cases mainly had severe low back pain, recent knee injuries or surgeries, or the presence of lateral knee OA.

### Demographic information

Demographic characteristics of forty-seven participants are presented in Table 1. Over 78% of the participants were females. Thirty (64%) participants had mild knee OA (KL 2: 6, KL 3: 24) while 17 had moderate knee OA. Participants with moderate knee OA were older in age and had higher BMI. These participants also showed greater knee varus angle and smaller early stance phase knee flexion excursion than those with mild knee OA.

**Table 1:** Descriptive characteristics of participants

Characteristics	All (*n* = 47)	Mild knee OA (*n* = 30)	Moderate knee OA(*n* = 17)	Difference between mild and moderate OA (*p*-value)
Demographic				
Age (year)	62.1 ± 6.0	60.7 ± 5.9	64.4 ± 5.8	0.052
BMI (kg/m^2^)	26.3 ± 3.6	25.4 ± 3.4	27.8 ± 3.3	0.019
Gender (female/male)	37/10	27/3	10/7	0.023
Pain (VAS)	5.2 ± 1.8	4.8 ± 1.7	5.8 ± 1.7	0.068
Walking speed (m/s)	1.0 ± 0.2	1.0 ± 0.2	0.9 ± 0.2	0.039
Alignment				
Knee varus angle (°)	6.7 ± 5.0	4.4 ± 3.1	10.7 ± 5.1	< 0.001
Tibial torsion (°)	26.5 ± 5.8	26.8 ± 5.9	25.9 ± 5.8	0.731
Lower limb kinematics				
Knee flexion at initial contact	16.33 ± 6.55	16.17 ± 5.79	16.61 ± 7.89	0.790
Peak knee flexion in early stance	21.07 ± 7.58	21.82 ± 6.80	19.73 ± 8.86	0.521
Knee flexion excursion in early stance	4.74 ± 2.92	5.65 ± 2.71	3.12 ± 2.63	0.004
Ankle dorsi-flexion at initial contact	1.27 ± 4.23	1.11 ± 4.22	1.56 ± 4.39	0.367
Peak ankle dorsi-flexion in early stance	2.55 ± 3.60	2.81 ± 3.70	2.10 ± 3.48	0.073
Ankle dorsi-flexion excursion	23.53 ± 5.05	22.94 ± 4.21	24.75 ± 6.21	0.092
Foot progression at initial contact	11.16 ± 7.16	11.42 ± 6.17	10.68 ± 8.84	0.492

^*^ denotes *p* < 0.05 between mild and moderate OA

### Interaction with OA severity

There was significant interaction of OA severity with tibial torsion in its relationship with knee flexion excursion in early stance phase (*p* = 0.001). Knee varus angle had statistically significant interaction with severity in the relationship with foot progression angle at initial contact (*p* = 0.047). No other group interaction was found as shown in Table 2.

**Table 2:** Interaction of OA severity between lower limb kinematics and alignment

	Tibial torsion				Knee varus angle			
	Coefficient	95%CI	*p*-value	Adjusted R^2^	Coefficient	95%CI	*p*-value	Adjusted R^2^
Knee flexion at initial contact	-0.13	-0.86, 0.59	0.717	0.05	-0.25	-1.29, 0.80	0.638	0.06
Peak knee flexion in early stance	-0.58	-1.39, 0.24	0.164	< 0.01	-0.40	-1.60, 0.79	0.501	0.03
Knee flexion excursion in early stance	-0.44	-0.70, -0.18	0.001*	0.31	-0.15	-0,56, 0.25	0.448	0.19
Ankle dorsi-flexion at initial contact	0.02	-0.45, 0.49	0.949	0.07	-0.02	-0.70, 0.66	0.945	0.06
Peak ankle dorsi-flexion in early stance	-0.16	-0.55, 0.24	0.429	0.04	-0.12	-0.70, 0.45	0.662	0.05
Ankle dorsi-flexion excursion	-0.17	-0.51, 0.16	0.305	0.01	-0.10	-0.59, 0.38	0.674	0.03
Foot progression at initial contact	-0/13	-0.85, 0.59	0.716	0.14	-1.10	-2.19, -0.01	0.047*	0.04

^*^Significant level = 0.05

### Relationship between joint alignment and lower limb kinematics during early stance phase

Table 3 shows correlation between joint alignments and gait kinematics. Larger knee varus angle was associated with smaller knee flexion excursion (r = -0.39, *p* = 0.010) in all participants. External foot progression angle was positively associated with larger external tibial torsion (r = 0.48, *p* = 0.001). The findings showed that participants with greater external foot progression angle had greater external tibial torsion.

**Table 3:** Association between knee sagittal kinematics and alignment in all participants

	Tibial torsion		Knee varus angle	
	r	*p* value	r	*p* value
Knee flexion at initial contact	< -0.01	0.988	-0.08	0.595
Peak knee flexion in early stance	< 0.01	0.990	-0.22	0.167
Knee flexion excursion in early stance	-	-	-0.39	0.010
Ankle dorsi-flexion at initial contact	0.05	0.760	0.01	0.958
Peak ankle dorsi-flexion in early stance	0.11	0.488	-0.06	0.725
Ankle dorsi-flexion excursion	0.06	0.698	-0.08	0.622
Foot progression at initial contact	0.48	0.001	-	-

Controlling for gender, age, BMI, speed, pain

### OA Severity specific relationship between joint alignment and lower limb kinematics during early stance phase

**Fig. 1:**
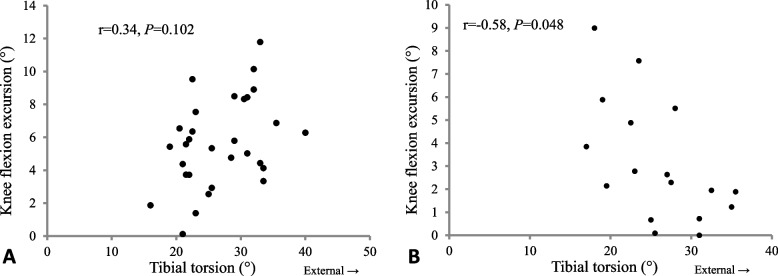
Scatter plot of relationship between knee flexion excursion and tibial torsion (**A** mild OA, **B** moderate OA)

Sub-group analysis indicated a significant negative association between external tibia torsion and knee flexion excursion in the moderate group (r = -0.58, *p* = 0.048; Fig. 1). Such observation could not be detected in those with mild knee OA (r = 0.34, *p* = 0.102). Hence, in individuals with moderate knee OA, greater knee flexion excursion was related to less external tibial torsion. No association was found between foot progression angle at initial contact and knee varus angle in the subgroup analysis.

## Discussion

The present study aimed to explore the relationships between tibial torsion and lower limb kinematics at early stance phase during gait. Findings from this study provide evidence that lower limb alignment was associated with early stance knee flexion excursion and foot progression angle in people with medial knee OA. Specifically, tibial torsion was related to foot progression angle in all participants, and it was also associated with early stance phase knee flexion excursion in those with moderate knee OA. Knee varus angle showed a negative association with knee flexion excursion.

The novice finding from this study was a negative relationship between external tibial torsion angle and knee flexion excursion during early stance phase of gait in participants with moderate knee OA. Such finding indicated that in participants with moderate OA severity, smaller knee flexion excursion was related to greater external tibial torsion. This association between tibial torsion and knee flexion excursion would support our initial assumption that torsional alignments would possibly affect knee motion in sagittal plane. This result was consistent with a previous study based on a computerized model that tibial torsion could possibly influence the positioning of knee flexion–extension axis in the horizontal plane [15]. With more external rotated tibia, the more proximal parts of lower limb could have more internal rotation to attempting a more neutral lower limb alignment. In the loading response phase, knee joint flexes in a close chain manner from nearly full extension. The external tibial torsion could disrupt the unlocking mechanism of knee joint for initiating flexion which requires external rotation of femur on tibia in the loading response phase of gait cycle in moderate OA. Loss of such mechanism and having inadequate knee flexion is harmful to knee joint, since it had been discussed that larger knee flexion at early stance phase can facilitate impact absorption and loads transmission [25, 26]. In our previous report, we found that higher external tibial rotation was associated with greater external knee adduction moment in early stance phase in moderate knee OA, which indicated possible overload of knee joint medial compartment because of the high external tibial torsion [27]. When external tibial torsion was more likely associated with smaller knee flexion excursion, the external tibial torsion might be a potential harmful factor to knee OA mechanically. In the meanwhile, the present study found this relationship was specific to radiographic severity of OA. It agreed with our hypotheses that the relationship would be different in the two subgroups, and this difference is potentially related to progressive structural destruction of knee joint in these patients. In the present study, the relationship between tibial torsion and knee flexion could not be established in mild knee OA. Knee flexion excursion was smaller in people in early OA than established OA [28], and smaller in moderate OA than more severe OA [29, 30]. The present study detected an 80% smaller knee flexion excursion in the moderate than the mild knee OA group. Therefore, a decreasing trend of knee flexion excursion could be proposed with an advance in OA severity from the mild to moderate stage. People with moderate knee OA suffered more from stiffened knee sagittal motion. Nevertheless, there was no significant difference of tibial torsion angle between these two subgroups in the present study. Given the fact that the sample size was relatively small, the reason why only subgroup of moderate OA showed a negative relationship between knee flexion excursion and external tibial torsion remains unclear. We speculated that knee joint deformation could be the reason for this relationship. The severer osteophytes and in moderate knee OA could possibly hinder the coupling mechanism between torsional angle and knee flexion–extension motion. Nonetheless, the OA severity specific correlation hinted that tibial torsion could be more influential in the kinetic chain of lower limb in these more severe patients. The more proximal part in the kinetic chain, knee joint was less functioned and left less freedom for compensatory motion during gait.

Our study also detected a significant association between external foot progression angle and external tibial torsion. Thus, greater external tibial torsion occurred more possibly in people walking with greater external foot progression angle. Torsional alignments of the lower limb were closely interacted to maintain a relatively neutral position [31]. It had been suggested that tibial torsion was a strategy to neutralize abnormal foot progression angle [32]. In healthy population, the amount of tibial torsion angle was observed to have a positive association with foot progression angle [33]. Findings from this study showed a similar relationship in participants with knee OA. In adult population, the foot progression angle is external rotated for approximately 13° [34]. With this reference value, 43.3% of and 35.3% of our participants with the mild OA and moderate OA walked with greater external foot progression angle, respectively. The gait pattern with a large external foot progression angle was known as toe-out gait. The toe-out gait modification was associated with reduction in knee flexion excursion during the early stance phase of gait [35]. Gait re-education, such as an adoption of a toe-out gait, has been suggested to people with knee OA, because a toe-out gait had a favorable effect on de-loading the medical compartment of knee joint comparing to natural gait by reducing peak external adduction moment which serves as a surrogate for medial-to-lateral knee loading [36, 37]; however, the reduction existed at the second half of stance phase but not in the early stance phase, according to the summarized information from a meta-analysis [6]. Therefore, the beneficial effect of the toe-out gait on de-loading knee joint in loading response phase remained uncertain. On the other hand, findings from present study indicated that a larger external foot progression angle toe-out gait might be associated with external tibial rotation; and increase in external tibial rotation might relate to reduced knee flexion excursion during the early stance phase in those with moderate knee OA. The adoption of toe-out gait training might have different effects due to disease severity. Further research on this topic is warranted to confirm whether a disease severity specific gait modification is needed.

Our study also detected those participants with greater knee varus angle had smaller early stance flexion excursions. This was consistent with a previous report that showed the early stance phase knee flexion excursion in people with knee OA with varus malalignment was significantly smaller than that of healthy control or people with knee OA without varus malalignment [38]. In addition, there was a negative association between medial joint space narrowing of knee and early stance phase knee flexion excursion in people with medial knee OA [39]. Since knee varus malalignment could cause narrowing of medial joint space [40], it might affect the knee flexion excursion in the early stance phase.

This study was a cross-sectional study; therefore, the causal relationship could not be established according to the design and finding of current study. Also, our findings cannot be generalized to participants with severe knee OA, those with KL grade 4. One of the limitations is that potential confounders, such as knee joint contracture, tendon tightness, foot position, etc., were not measured. Further study could be designed to explore the change of foot progression angle on knee flexion excursion during gait in people with different OA severity.

## Conclusions

We concluded that tibial torsion was related to early stance knee flexion excursion and foot progression angle during gait in people with medial knee OA. More specifically, external tibial torsion was associated with a smaller stance phase knee flexion excursion in moderate knee OA, and it was identified as a factor related to external foot progression angle. Such findings suggest that lower limb alignments in both horizontal and frontal planes would influence knee and foot kinematics during stance phase of gait. This OA severity specific relationship provided a new clue for future study to investigate a different gait modification strategy in people with moderate knee OA of excessive external tibial torsion.

## Data Availability

The datasets used and/or analyzed during the current study are available from the corresponding author on reasonable request.

## Abbreviation

BMI: Body mass index
ICC: Intraclass correlation coefficient
KL: Kellgren-Lawrence grading scale
OA: Osteoarthritis
VAS: Visual analogy scale
